# A cluster randomized controlled trial to assess the impact of the ‘Caring for Providers to Improve Patient Experience’ intervention on person-centered maternity care in Kenya and Ghana: Study Protocol

**DOI:** 10.21203/rs.3.rs-4344678/v1

**Published:** 2024-05-10

**Authors:** Patience A. Afulani, Monica Getahun, Linnet Ongeri, Raymond Aborigo, Joyceline Kinyua, Beryl A. Ogolla, Jaffer Okiring, Ali Moro, Iscar Oluoch, Maxwell Dalaba, Osamuedeme Odiase, Jerry Nutor, Wendy Berry Mendes, Dilys Walker, Torsten B. Neilands

**Affiliations:** University of California San Francisco; University of California San Francisco; Kenya Medical Research Institute; Navrongo Health Research Centre; Kenya Medical Research Institute; Global Programs for Research and Training; Global Programs for Research and Training; Navrongo Health Research Centre; Migori County Government; University of Health, and Allied Sciences (UHAS); University of California San Francisco; University of California San Francisco; Yale University; University of California San Francisco; University of California San Francisco

## Abstract

**Background:**

Poor person-centered maternal care (PCMC) contributes to high maternal mortality and morbidity, directly and indirectly, through lack of, delayed, inadequate, unnecessary, or harmful care. While evidence on poor PCMC prevalence, as well as inequities, expanded in the last decade, there is still a significant gap in evidence-based interventions to address PCMC. We describe the protocol for a trial to test the effectiveness of the **“Caring for Providers to Improve Patient Experience” (CPIPE)** intervention, which includes five strategies for provider behavior change, targeting provider stress and bias as intermediate factors to improve PCMC and to address inequities.

**Methods:**

The trial will assess the effect of CPIPE on PCMC, as well as on intermediate and distal outcomes, using a two-arm cluster randomized controlled trial in 40 health facilities in *Migori* and *Homa Bay Counties* in Kenya and *Upper East* and *Northeast Regions* in Ghana. Twenty facilities in each country will be randomized to 10 intervention and 10 control sites. The primary intervention targets are all healthcare workers who provide maternal health services. The intervention impact will also be assessed first among providers, and then among women who give birth in health facilities. The primary outcome is PCMC measured with the PCMC scale, via multiple cross-sectional surveys of mothers who gave birth in the preceding 12 weeks in study facilities at baseline (prior to the intervention), midline (6 months after intervention start), and endline (12 months post-baseline) (N = 2000 across both countries at each time point). Additionally, 400 providers in the study facilities across both countries will be followed longitudinally at baseline, midline, and endline, to assess intermediate outcomes. The trial incorporates a mixed-methods design; survey data alongside in-depth interviews (IDIs) with healthcare facility leaders, providers, and mothers to qualitatively explore factors influencing the outcomes. Finally, we will collect process and cost data to assess intervention fidelity and cost-effectiveness.

**Discussion:**

This trial will be the first to rigorously assess an intervention to improve PCMC that addresses both provider stress and bias and will advance the evidence base for interventions to improve PCMC and contribute to equity in maternal and neonatal health.

## BACKGROUND

Maternal mortality and morbidity remains very high in sub-Saharan Africa (SSA), despite progress in the last decade.^1^ The estimated maternal mortality ratio for SSA is at 546 per 100,000 live births—with Ghana and Kenya at 308 and 510, respectively, compared to about 12 in high-income regions.^1^ Further, for every woman who dies, about 20 others suffer from various morbidities.^2^ These outcomes occur alongside poor fetal outcomes including stillbirths, prematurity, and early neonatal deaths. High quality care during childbirth is critical for preventing maternal and neonatal mortality, given roughly three-quarters of maternal and fetal deaths occur from complications during labor, delivery, and the first 24 hours postpartum.^3^ While complications are difficult to predict, they can effectively be managed and deaths averted, when recognized and treated promptly.^3,4^ Thus, skilled care by health professionals who can identify and treat complications and provide basic care and referrals, are essential at every delivery.^3^ Until recently, most efforts to improve maternal and child health (MCH) outcomes in SSA focused on increasing the use of MCH services. While countries in SSA, such as Ghana and Kenya, report increasing coverage for facility births, significant gaps in equity and quality of care persist between and within countries.^5^ For instance, although about 70% of births in SSA occur in health facilities, wide disparities in facility-based births persists, especially by SES.^6^ Further, poor quality care in many health facilities, coupled with the lack of reduction in mortality on par with increased facility deliveries, has called attention to the quality of care.^7–9^ Quality of care includes both service provision and experience of care.^10^ Most efforts have, however, emphasized service provision—the technical aspects of quality of care; fewer address experience of care—the person-centered dimensions and vehicle by which care is delivered.^11^

Person-centered maternity care (PCMC) is a key component of quality of care that captures the interpersonal aspects of care and impacts patient experience. It emphasizes maternal health care that is respectful, compassionate, and responsive to individual patient preferences, needs, and values.^12^ Key domains of PCMC include dignity and respect, communication and autonomy, and supportive care. PCMC is a critical component of a human rights framework, as everyone has a right to be treated with dignity and respect, including during pregnancy and childbirth.^13–16^ However, globally, studies have shown significant gaps in PCMC as evidenced by disrespect and abuse, poor communication, lack of respect for women’s autonomy, and lack of supportive care during prenatal care and childbirth.^17–20^ Such poor PCMC contributes to both the low rates of facility-based deliveries and the disparities,^21,22^ with the poorest outcomes among the most vulnerable, who are more likely to be mistreated and stigmatized in health facilities.^18,19,23,24^ Further, PCMC contributes to improved health outcomes through timeliness, patient engagement, safety, improved psychosocial health, and patient and provider satisfaction.^25,26^ Recent research also shows PCMC domains are associated with improved maternal and neonatal outcomes such as shorter duration of labor, decreased caesarean and instrumental vaginal birth, higher five-minute Apgar scores, early postnatal care, breastfeeding, and lower risk of screening positive for post-partum depression.^27–32^ Poor PCMC therefore undermines health gains for mothers and babies.^33^

Several studies have highlighted the multilevel drivers of poor PCMC, which include inadequate provider knowledge of PCMC, poor provider attitudes, provider stress, burnout, and bias, power asymmetry between patients and providers, lack of accountability mechanisms, institutional and health system factors, and broader social and gender norms that facilitate and normalize disrespect and abuse.^34–41^ There is, however, still limited research on interventions to improve PCMC in LMICs, with most interventions focused on individual drivers such as provider knowledge.^42,43^ Further, existing interventions in LMICs do not address the role of provider stress, burnout, and difficult patient-provider interactions in PCMC, nor do they explicitly address the inequities in PCMC.^38,44–46^ To address the gap in evidence-based interventions to improve PCMC, we designed the “Caring for Providers to Improve Patient Experience” (CPIPE) intervention to address drivers of poor PCMC and center the unique needs of vulnerable women in LMICs. The development of the intervention has been previously described in detail.^47^

**Figure 1:**
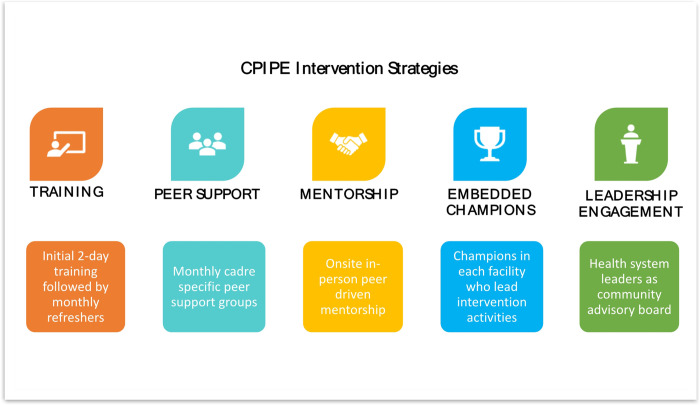
CPIPE Intervention strategies

CPIPE is a theory and evidence-based intervention that was designed through an iterative process of formative research and feedback from stakeholders, and informed by the Ecological Perspective,^48^ Social Cognitive Theory,^49^ and Trauma Informed System framework.^50^ It leverages 5 strategies: provider simulation-enhanced training, peer support, mentorship, embedded champions, and leadership engagement (Fig. 1). The training, which is delivered through short didactic, interactive training, and reflective sessions over 2 days, includes content on PCMC, stress management, dealing with difficult situations, and implicit and explicit bias. This content is integrated into customized highly realistic emergency obstetric and neonatal care (EmONC) simulations and teamwork and communication activities designed in collaboration with PRONTO International to enable providers to apply concepts in the context of an emergency scenario with guided self-reflection through debriefing.^51^ The two-day training is followed by monthly refreshers for six months, as well as resources shared on WhatsApp to reinforce training content over 6 months. Peer support groups are cadre-specific groups of the providers that are facilitated monthly by a peer leader to debrief on events at the maternity unit and brainstorming solutions; and engaging in stress management activities. Mentorship involves intentional mentor-mentee pairing based on a survey of mentor and mentee needs and preferences to facilitate transfer of knowledge and skills through one-on-one relationships. Embedded champions are selected by their peers in each facility to lead intervention activities in their facility, including facilitating the monthly refreshers and peer support groups. As part of our leadership engagement, a Community Advisory Board (CAB), with representatives from the county/regional, facility, as well as providers and patients, guides the implementation and serves as a body for high-level advocacy. The 2-day training aims to increase provider knowledge, skills, and self-efficacy to impact attitudes and behaviors towards preventing burnout, mitigating bias, and improving PCMC while the additional strategies create an enabling environment for behavior change.

A pilot study in Kenya showed the CPIPE intervention has high feasibility, acceptability, and preliminary effectiveness on provider stress, burnout, bias, and provider-reported PCMC provision^20,52^. Thus, the next step is to assess the effectiveness of *CPIPE* on patient-reported measures and to explore *CPIPE’s* mechanisms of action in a rigorously designed and fully powered study. This paper describes the protocol for this next phase of our work.

## METHODS

### Aims

**Figure 2:**
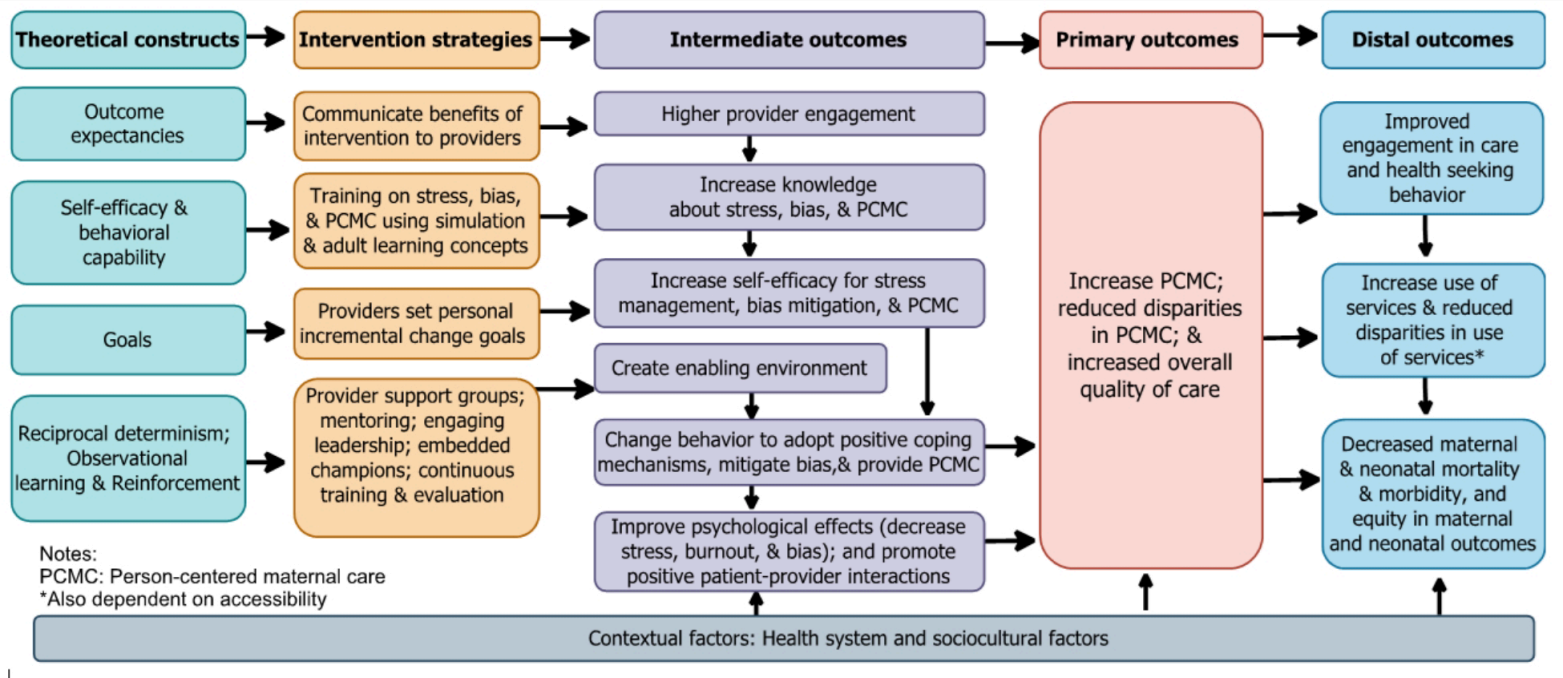
CPIPE conceptual framework

Our specific aims, as shown in Fig. 2, are to:

1. *Assess the effectiveness of the CPIPE intervention on PCMC*. We hypothesize that *CPIPE* will improve PCMC for all women, but especially for low SES women. In addition, we will conduct a cost-effectiveness analysis of CPIPE on PCMC.
2. *Examine the mechanisms of impact of CPIPE.* We hypothesize that CPIPE will improve intermediate outcomes (provider knowledge and self-efficacy, stress, burnout, and bias levels), which will in turn impact PCMC. We will assess the effect of the intervention on these intermediate outcomes and examine if changes in these outcomes account for the effect of *CPIPE* on PCMC. We will also assess implementation outcomes including fidelity and quality of implementation that may contribute to differential effects.
3. Assess impact of the CPIPE intervention on distal outcomes. This is an exploratory aim in which we will examine if the intervention impacts more distal outcomes in our conceptual framework, including timely postnatal care, breastfeeding initiation and exclusivity, postpartum mental wellbeing, neonatal complications, and maternal and neonatal mortality; and if changes in PCMC account for these effects.

### Study design and setting

We plan to test the effectiveness of the *CPIPE intervention* on PCMC and intermediate and distal outcomes using a two-arm cluster randomized controlled trial (RCT) in 40 high volume health facilities in Migori and Homa Bay Counties in western Kenya and in the Upper East and Northeast Regions of northern Ghana. The counties and regions in each country were selected for having among the worst maternal and neonatal health outcomes, having similar characteristics, and based on our existing institutional relationships. Migori and Homabay are neighboring, and similar counties located along Lake Victoria in in western Kenya that have comparable maternal and neonatal mortality burden. They have 8 sub-counties, each with a sub-county hospital and one county referral hospital. There are about 155 and 263 health facilities in Migori and Homa Bay, respectively, including county and sub-county hospitals, health centers, faith-based, and private health facilities.^53^The Upper East and North East Regions are also neighboring regions located in the north-eastern corner of Ghana, both sharing borders with Togo to the east that also have similar characteristics and maternal and neonatal mortality burden. The Upper East Region shares boundaries with Burkina Faso to the north and Northeast Region to the south. The Upper East region is divided into 15 districts, with 11 district hospital, 67 health centers, 419 CHPS compounds and one regional hospital that serves as a referral center for the district hospitals.^54,55^Northeast is divided into six districts, with five district hospitals, 21 health centers, and 154 CHPS compounds. ^56^

### Randomization

**Figure 3:**
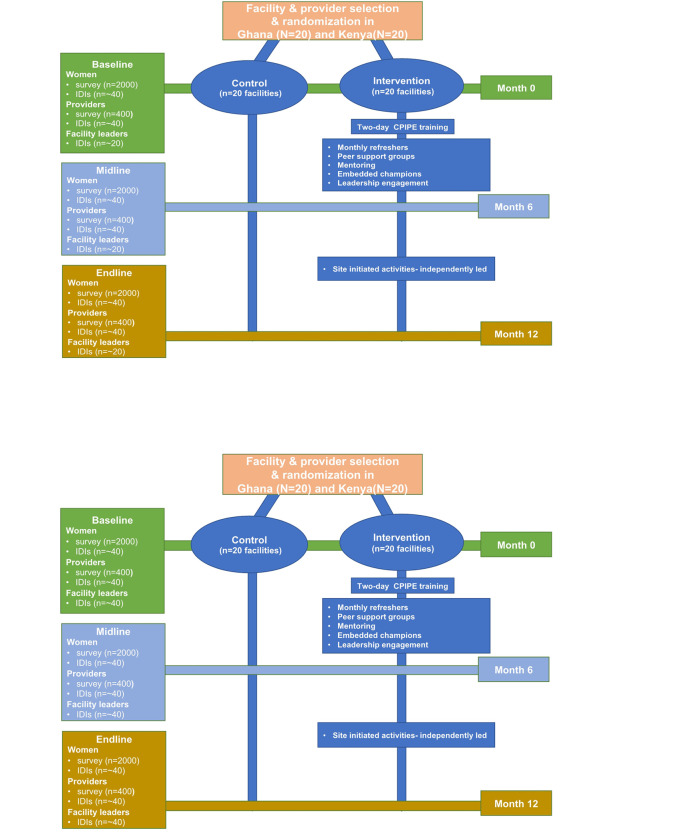
CPIPE study design and randomization

Randomizing individual providers is not ideal because of a high potential for contamination within facilities and goal of changing facility culture. A cluster RCT design allows us to address threats to internal validity and account for natural clustering of providers within facilities.^57^ Forty facilities will be randomized to intervention (N = 20) and control (N = 20), with arms stratified by country(10 intervention and 10 control facilities in each country). To reduce the risk of contamination due to interaction of providers within the same sub-counties and districts, randomization will be at the sub-county/district level such that control and intervention facilities are not in the same sub-county/district. The intervention group will receive all strategies of the *CPIPE* intervention strategies over a period of 6 months after the baseline data collection. At the end of the 6-month implementation, we will collect midline data. Facilities will then be encouraged to continue with the intervention activities without involvement of the study for an additional 6 months, with final data collection at 12-months (endline). The control group will not receive the CPIPE intervention during the 12-month data collection period but will maintain their usual facility level activities. We plan to implement the intervention in the control sites after the endline data collection (assuming preliminary effectiveness). The protocol complies with the standard protocol items for cluster randomized trials. The study design, outcomes, and participant flow are summarized in Figs. 2 and 3.

### Study population

The study population includes providers and women who give birth in the study facilities. Providers are the recipients of the intervention as well as potential beneficiaries. All healthcare workers (including nurses, midwifes, doctors, clinical/medical officers, and support staff) who provide MCH services (including antenatal, intrapartum, and postnatal care) in study facilities will be eligible for the intervention. Including all providers in a facility will facilitate an enabling facility culture, across all cadres. Women who receive care in the study facilities are the anticipated beneficiaries of the intervention; we will obtain data on the primary outcomes among women. Study participants will be women who gave birth in the facility in the 12 weeks preceding data collection. Other eligibility criteria are shown in table 1.

### Study procedures

#### Intervention

This will include participation in an initial 2-day training, followed by monthly refreshers to reinforce training content; peer support groups, each comprising 5–10 providers of similar cadre, who will meet for about 1–2 hours every month; and in a mentorship program, in which mentor/mentee pairs are encouraged to meet once a month but will be primarily mentee-driven. Each facility will have two embedded champions, providers nominated by their peers who will lead activities at each site including facilitating refreshers and peer support groups. Leadership engagement will occur throughout the project through continuous interaction with leaders in the study sites and with a community advisory board which will meet quarterly to provide input on implementation and respond to provider concerns to address sources of stress.

#### Data collection

We will collect data at 3 time points in the intervention and control sites: baseline (T1) prior to the intervention start at each facility, midline 6 months post baseline/intervention start (T2) to assess immediate impact, and endline 12 months after baseline (6 months post intervention) (T3) to assess sustainability. Survey data will be collected utilizing Redcap programmed tablets. Process data will be collected throughout the study to assess intervention fidelity and implementation using study logs and Redcap. The provider cohort will be followed longitudinally for 12 months following baseline enrollment. The women’s sample will be multiple cross-sections, where data will be collected from 3 different groups of women at the 3 time points. We will use a mixed-methods approach. Quantitative data will be obtained from surveys followed by IDIs to qualitatively explore factors that may influence outcomes. We will also assess intervention fidelity through observations of intervention activities and review of project logs.^58,59^

#### Sample size

Participants include 400 providers (N = 400) followed longitudinally and N = 6000 women participating in multiple cross-sectional surveys-baseline (N = 2000), midline (N = 2000), and endline (N = 2000)-across both countries: i.e., 200 providers followed longitudinally, and 3000 women interviewed once across 3 time points (N = 1000 at each time-point) from both intervention and control facilities, in each country. We will then conduct IDIs with a subset of providers (N = ~ 40), facility leaders (N = ~ 40), and mothers (N = ~ 40) to explore pathways to intervention outcomes. Power analysis for sample size justification is described under the analysis section.

#### Recruitment

The study will first be introduced to the county/region, sub-county/district, and facility leadership, who will inform all providers who work in MCH units in the study facilities. The study team will then approach individual providers to provide additional information and obtain written informed consent. Only providers who provide individual informed consent will be enrolled. Providers will be recruited and consented at the beginning of the study and then followed longitudinally for the three rounds of data collection. Women will be recruited at health facilities following discharge from the facilities and after postnatal care and in the immediate communities served by the study facilities. They will first be identified with the help of health care providers, and community health volunteers and review of facility birth registers, at the study sites. The study team will then screen them for eligibility, provide information about the study, and obtain individual informed consent if interested. Only women who provide written consent will be interviewed either at the facility, at their homes, or at a preferred location. Following surveys, all participants will be asked if they are willing to be contacted for a follow-up IDIs; a subset of those who consent will be recontacted for the IDIs. Participants for IDIs will be purposively sampled balancing for age, time/weeks since birth, and post-natal care attendance, to represent the range of experiences. All women will be consented prior to IDIs.

### Retention

Because providers will be followed longitudinally several steps have been planned to reduce attrition. First, providers who plan to leave the study within the study period will not be enrolled at baseline. Second, we will discuss with facility and health system leaders in the study sites to reduce rotation and transfer of providers during the study period unless necessary. Third providers will receive an incentive for participation in all implementation and research activities. Attrition is not a concern for women since they will not be followed longitudinally.

### Study outcomes

#### Aim 1: The primary outcome for the study is PCMC to assess the effectiveness of the CPIPE intervention on PCMC.

PCMC will be measured with the PCMC scale developed and validated by our team in Kenya and Ghana.^60–62^ The PCMC scale is a 30-item scale administered to perinatal women with 3 sub-scales for dignity and respect, communication and autonomy, and supportive care. Items for each scale are summed to create a score, which is standardized to range from 0 to 100, where higher scores indicate more person-centered care.

#### Aim 2: Secondary outcomes to examine the mechanisms of impact of CPIPE on PCMC include

provider stress and burnout; bias awareness and mitigation; as well as provider knowledge, self-efficacy, and behaviors. Provider stress and burnout will be measured using the Cohen perceived stress scale ^63^ and the Shirom-Melamed Burnout measure,^64^ which we successfully used in Ghana and Kenya with demonstrated good psychometric properties.^46,65^ Other outcomes for this aim include provider knowledge, self-efficacy, and behaviors related to stress, bias, and PCMC. These will be measured using validated measures of stress^63^ and burnout,^64^ which we successfully used in Ghana and Kenya with demonstrated good psychometric properties,^46,65^ as well as tools we developed from the evaluation of the pilot study. For implicit and explicit bias, we will use the Bias Awareness and Mitigation in Maternal Health scale^66^ and vignettes, developed and tested in our prior research.^67^ We will measure provider knowledge, self-efficacy, and behaviors related to stress, bias, and PCMC, using tools developed for the evaluation of the pilot study. Longitudinal provider surveys will be used to collect this data in all study arms at baseline, midline, and endline.

#### Aim 3: Outcomes to Assess effect of the CPIPE intervention on distal outcomes in our conceptual framework

(Fig. 2) include receipt of timely postnatal care, breast feeding initiation and exclusivity, postpartum mental wellbeing, and post-delivery neonatal complications, which we have shown are associated with PCMC.^29,30^ These will be measured using questions on timing of breast feeding onset and current breastfeeding practices, the Edinburgh postnatal depression scale,^68^ and other questions on postpartum and newborn health in the women’s survey. We will also collect facility-level data on coverage indicators and maternal and neonatal morbidity and mortality from facility records. All study outcomes and sample measures are shown in Table 2. As part of all surveys, we will collect data on various covariates shown in Table 2.

### Analysis plan

#### Data quality assurance, initial analyses, and missing data.

We will use Redcap to perform real-time checks for data quality assurance.^69,70^ We will use frequency tables and measures of central tendency and variability for continuous variables to characterize the sample overall and by randomization group. If the two groups differ significantly at baseline on one or more covariates, we will use methods based on the Rubin causal model (e.g., propensity scores, double-robust estimation) to obtain the desired effect estimates under the counterfactual assumption of balanced groups.^71,72^ We will address incomplete data with direct maximum likelihood (ML) and multiple imputation (MI)^73^ because they make the relatively mild assumption that incomplete data arise from a conditionally missing-at-random (MAR) mechanism.^74^ Auxiliary variables will be included to help meet the MAR assumption.^75^ The proposed analyses will be conducted using STATA All program code and results will be documented extensively and archived to enable future review, transparency, and results reproducibility.

#### Aim 1:

We hypothesize that, mothers in the *CPIPE* intervention group will have higher mean PCMC scores than mothers in the control group at 6 months (H1a) and 12 months (H1b) after baseline. To test hypotheses H1a-H1b, in primary analyses we will fit two-level linear mixed models (LMM) to the PCMC score. Each of these two models will include a fixed effect for study arm with random intercepts for facility ID to account for clustering of women within health facilities. Because we will compare *CPIPE* to control at 6 months and then repeat the same comparison at 12 months, alpha (α) will be set at .05/2 = .025 for each of these two planned comparisons. In secondary analyses for Aim 1 we will extend the LMMs for PCMC to include effects for age and SES and their interactions with the intervention group indicator to explore whether the *CPIPE* intervention differentially benefits younger and lower SES women. Sex as a Biological Variable is not applicable: all participants will be biological females.

#### Aim 1 Power Analysis:

We used the NCSS PASS^76^module for a two-level multilevel model with a continuous outcome and randomization at the cluster (i.e., health facility) level to compute the minimum detectable effect size estimates for hypotheses H1a-H1b. We assumed power = .80, α = .025 per comparison, and N = 2000 women from 40 facilities. We further assumed ICC = .16 based on our previous data.^20,77^ The minimum detectable standardized mean difference was *d* = .41, which is between a small and medium effect size, suggesting the proposed primary analyses have sufficient power to detect small to medium effects.^78^

#### Aim 2

We anticipate that the intervention will positively impact the intermediate outcomes in our conceptual model (Fig. 1), leading to an improvement in PCMC. We thus hypothesize that, following *CPIPE* exposure, intervention providers will have, higher mean scores on provider knowledge (H2a); self-efficacy (H2b); enabling environment (H2c); behavior (H2d), and lower mean scores on work-related psychological effects (stress, burnout, and bias) (H2e) relative to control providers. To test these hypotheses, we will fit three-level LMMs to each intermediate outcome, with fixed effects for study arm, time, and their interaction, random intercepts for facility ID to account for clustering of providers within health facilities, and random intercepts, random slopes, and their covariance for person ID to account for clustering of repeated measurements within providers. We will perform time-averaged comparisons of repeatedly measured post-baseline observations of the key intermediate outcomes across study arms to examine *CPIPE* intervention effects.

In secondary exploratory analyses, we will investigate whether the intermediate outcomes measured for providers at 6 months mediate the relationship between *CPIPE* intervention assignment and PCMC at 12 months. These analyses will be conducted using principles of structural equation modeling (SEM) and causal mediation methods.^1^We will explore whether sex as a biological variable differentially affects intervention effects on the provider-level primary analyses by adding a sex main effect and a sex-by-*CPIPE* assignment interaction term to the LMM models. Sex as a biological variable is not applicable for the mediation analyses due to the outcome applying to women only. Aim 2 Power Analysis: We used the NCSS PASS^76^module for three-level multilevel models with randomization at the cluster level to compute the minimum detectable effect size estimates for hypotheses H2a-H2e. We assumed power = .80, α = .05, and 2 post-baseline repeated assessments from N = 400 enrolled providers from 40 health facilities (N = 320 following 20% conservatively assumed attrition based on pilot data and our planned approach to reduce attrition).^79^ We conservatively assumed ICC = .15 based on our preliminary data.^46,67,77^ Since the within-provider correlations are unknown, we varied them from r = .20 (small) to *r* = .80 (large). We computed the minimum detectable standardized mean difference*d* using the same inputs as listed above, yielding *d* = .40 to .44, which are between thresholds for small (.20) and medium (.50) standardized effects, suggesting the sufficient power to detect small to medium effects.^80^

#### Aim 3

Improvements in PCMC are hypothesized to impact timely and appropriate care provision, care engagement, future health seeking, and the physical and psychosocial health of women and their babies who receive care from those facilities, all of which positively influence maternal and neonatal outcomes. Recognizing that these distal outcomes are influenced by broader contextual factors and take longer to change, we propose an exploratory third aim to assess impact of *CPIPE* on these outcomes during the study period, and whether they are mediated by changes in PCMC. Given the exploratory nature of this aim, initial analyses will be descriptive, with frequency tables for all distal outcome and measures of central tendency and variability for continuous distal variables at 6 and 12 months for intervention and control sites.

While facility data will be limited to N = 40 clinics, inferential analyses will be performed with N = 2000 women on distal outcomes. We focus on the key outcome of receipt of timely post-natal care. We hypothesize that among mothers giving birth in the study facilities, mothers who received care in the *CPIPE* intervention group will be more likely to receive postnatal care within 48 hours than mothers in the control group at 6 months (H3a) and 12 months (H3b) after baseline. To test H3a and H3b, we will fit two-level generalized linear mixed models (GLMM) to the binary timely postnatal care variable collected at 6 months and at 12 months. Each model will include a fixed effect for study arm and random intercepts for facilities for clustering of women within health facilities. Because we will compare *CPIPE* to control at 6 months and at 12 months, α will be set at.05/2 = .025 for each comparison. We will use the same GLMM approach to test the effects of *CPIPE* on other distal outcomes. Additional secondary exploratory analyses for Aim 3 will explore whether the PCMC scores mediate the relationship between *CPIPE* intervention exposure and receipt of timely post-natal care. As in specific Aim 2, we will perform mediation analyses using SEM and causal mediation methods.^81^ Sex as a Biological Variable is not applicable because all care recipients will be biological females.

#### Aim 3 Power Analysis.

We used a similar approach as for Aim1 to compute the minimum detectable effect size estimates for hypotheses H3a-H3b, assuming a broad range of 24–81% of women receiving timely post-natal care and ICC = .039 at the facility level based on our previous data.^20,77^ Minimum detectable raw proportion differences were 8.3–10.7%, which correspond to small to medium effects.^80^

### Qualitative analysis

The sample size for the qualitative study is informed by guidelines to achieve data saturation^82,83^ and a desire to purposively sample from all the intervention facilities. We will record interviews and transcribe audio-recordings into English, translating if interviews are conducted in local languages. Transcripts will be coded and analyzed in Dedoose software utilizing collaboratively developed coding frameworks and group approaches to qualitative analyses, guided by the thematic analysis approach described by Braun and Clark.^84^ To capitalize on the mixed-methods design, findings from the qualitative and quantitative data collection and analyses will be integrated at the interpretation and discussion stages. All team members who collect the data will be included in the analyses to strengthen the validity of findings.

### Cost effectiveness analysis

We will also assess the cost-effectiveness of CPIPE on PCMC. We will use standard micro-costing techniques and include a full costing approach, which requires the estimation of both recurrent and capital costs related to the intervention. The recurrent costs will include cost of materials used within a year which will include cost on personnel salaries/allowances, medical consumables, training and meetings, travels, office supplies and overhead/administrative costs related to the intervention development and delivery. The capital costs will be the inputs or resources that usually last for more than one year, which includes items such as facility/office space, equipment, and vehicles/motorbike/bicycle costs. Research costs will be excluded. Capital costs will be annualized using a discount rate of 3% and useful life of the capital items. The total economic costs of the intervention will be estimated and then categorized into pre-intervention deployment costs, intervention implementation costs, and indirect costs.

#### Effectiveness:

The effectiveness outcome will be the PCMC scores measured for women at 6 months and at 12 months.

#### Cost effectiveness analysis:

The incremental cost–effectiveness ratio (ICER) will be used in the estimation of the cost-effectiveness of the CPIPE intervention. The ICER is the incremental cost per incremental benefit due to in CPIPE intervention. A budget impact analyses will be carried out to determine the impact of the intervention implementation and scale up on government budget.

#### Process evaluation

We will conduct a process evaluation to assess implementation and intervention fidelity including adherence, exposure, quality of delivery, competence, participant responsiveness, and program differentiation.^58,59^ We will use mixed-methods approaches for this purpose, guided by Proctor’s framework.^85,86^ A fidelity monitoring tool with structured and open ended questions will be used to document all implementation activities, noting details such as timing of activities, participants, facilitators, content of the activity, what went well and what did not go well. We will also document ongoing activities at each site to identify activities that might affect the outcomes of the trial.

#### Timeline

The CPIPE trial will be completed over a 5-year period. We will use the first 6 months for study preparation activities, including finalizing the protocol and data collection tools, updating the curriculum with lessons learned from the pilot, and obtaining ethical and other regulatory approvals at UCSF and all sites. We will implement the intervention in 2 blocks in each country, with 10 facilities (5 intervention and 5 control) in each block. Baseline data collection for the first block will start in Y1Q3, followed by intervention implementation, midline, and endline over a period of about 1 and half years. Baseline and intervention activities in the next block will start in about Y2 following the same approach in the next year. In year 3 we will offer the intervention to the control groups. Data collection across all sites is anticipated to be completed in Y4. Data cleaning, quality assessment, and preliminary analysis will occur as data are being collected. In year 5, we will continue with data analysis and preparation of manuscripts and presentations, with dissemination in Ghana and Kenya and globally.

## Discussion

The proposed intervention will be among the first, if not the first, rigorously designed and evaluated intervention to improve PCMC that addresses key drivers of poor PCMC, including provider stress and bias. The results will significantly advance the evidence base for interventions to improve PCMC and contribute to equity in maternal and neonatal health. A limitation of the *CPIPE* intervention is inability to address structural factors such as shortage of health workers and supplies that affect provider stress and PCMC. These are important, but beyond the scope of one intervention. This intervention is intended to help providers cope and provide better care amid the structural challenges. However, by collecting data on these issues and engaging leadership, we will provide the data and platform to prioritize and address structural challenges, and inform policy decisions at the county, regional, and national levels. Additionally, the problem-solving strategies identified during the intervention period may help address and advocate for some of these structural challenges. While recruitment and retention of providers may be a challenge, we will use lessons from the pilot studies, where we have achieved our target sample sizes, even during the COVID-19 pandemic. We have longstanding relationships and collaborations with health systems leaders in the study counties/regions, which will be critical to our success. In addition, our prior work has indicated both high need and interest in this intervention in the study areas.^20,52^ To reduce attrition, we will work with the county leadership to minimize transfer of providers during the study period where possible. To mitigate challenges with recruitment of women, we will utilize lessons from our prior studies such as working with community health volunteers and providing small incentives to both the women and volunteers who facilitate their recruitment. Other potential challenges include contamination from other related interventions in the study areas. As part of our process monitoring, we will document ongoing activities at each site to help explain unexpected findings.

Integrated provider targeted interventions that give providers the opportunity to learn, practice, and reflect on their own as well as the patient experience with attention to the needs of the most vulnerable, and which create an enabling environment for behavior change, have the potential to improve both the providers’ and women’s maternal experiences. Incorporating such interventions into in-service professional development programs will help advance global efforts to promote PCMC. If the intervention is proven to be effective, it will be disseminated to the Kenyan and Ghana Ministries of Health, as well as non-government stakeholders. We anticipate expanding this work elsewhere in SSA and working to develop guidelines for scaling similar interventions. This research will address a major gap in efforts to improve MCH outcomes and achieve equitable maternal and neonatal health.

## Figures and Tables

**Table 1:** Eligibility criteria

Eligibility criteria for providers
1. Working in the maternal health units of study facilities for at least 6 months at the time of data collection.
2. Capable and willing to provide informed consent.
3. Able and committed to attending the intervention training and follow up activities.
4. Age 18 or above.
Exclusion criteria for providers
Intend to leave within the study facility/catchment during the 6-month intervention period.
Eligibility criteria for women
1. Gave birth at the study facilities.
2. Gave birth within the 12 weeks preceding the data collection.
3. Capable and willing to provide informed consent.
4. Age 15 or above, with individuals aged 15–17 meeting the criteria for emancipated minors
Exclusion criteria for providers
Too ill to participate or do not live within a feasible distance/location if interviews are scheduled to be conducted in the community.

**Table 2:** Study outcomes, measures, sources, and timing of capture

		Measures/Tools	Sources/Data Level			Time period		
			Women	Providers	Facility	T1	T2	T3
**Primary outcome**	**Person-centered maternity care**	*PCMC Scale*						
**Secondary outcomes**								
**Intermediate Outcomes**	**Provider Knowledge**	*Knowledge of stress, bias & PCMC*						
	**Provider self-efficacy**	*Self-efficacy for stress management, bias mitigation & PCMC*						
	**Provider behavior**	*Implementation stress management, bias mitigation activities & PCMC activities*						
	**Enabling environment**	*Support from leadership, superiors, colleagues*						
	**Burnout**	*Shirom-Melamed Burnout scale* ^64^						
	**Perceived stress**	*Cohen perceived stress scale* ^63^						
	**Explicit bias**	*Vignettes on perceptions of different SES patients*						
	**Implicit Bias**	*Bias awareness and mitigation in maternal health scale ^112^*						
***Distal outcomes***	**Health seeking**	*Postnatal care within 2 days of birth for mother & baby*						
	**Health engagement**	*Time of initiation of breastfeeding, current breastfeeding & exclusive breastfeeding*						
	**Mental health**	*Edinburgh postnatal depression scale* ^68^						
	**Mortality/morbidity**	*Facility maternal mortality, Intrapartum stillbirth, neonatal mortality, key complications*						
**Covariates**	**Sociodemographic characteristics**	*Age, marital status, ethnicity, religion Household wealth, educational attainment, etc.*						
	**Obstetric history**	*Parity; past pregnancy, birth, & infant complications*						
	**Health status**	*Self-rated health, chronic & pregnancy-related comorbidities*						
	**Antenatal care**	*Antenatal care initiation, timing, frequency, & location*						
	**Intrapartum care**	*Gestational age, delivery mode, type of birth attendant*						
	**Sociodemographic characteristics**	*Age, gender, marital status, parity, position & years of experience education, income, etc.*						
	**Health**	*Self-rated health, chronic disease, depression, exercise,*						
	**Job demands & resources**	*No. of workdays per week & work hours per day; perceived overcommitment; traumatic work experiences; etc.*						
	**Facility climate**	*Perceived disrespect from supervisors, colleagues,*						
	**Designation**	*Facility type & level*						
	**Volume & resources**	*Total deliveries, no. of providers; caesarian section capability, Availability of essential commodities*						
**Process Eval**	**Fidelity**	*No. of providers engaged, trainings conducted, peer support groups formed, mentor mentee pairs, etc.*						
	**Quality**	*Perceptions about various activities, assessment of relevance, satisfaction, suggestions, etc.*						

## Data Availability

Data sharing not applicable as no datasets generated and/or analyzed for this paper.
